# Editorial: The role of trafficking in synaptic development, maintenance, and plasticity

**DOI:** 10.3389/fncel.2024.1362676

**Published:** 2024-01-10

**Authors:** Frederic J. Hoerndli, Peri T. Kurshan, Victor Anggono

**Affiliations:** ^1^Department of Biomedical Sciences, Colorado State University, Fort Collins, CO, United States; ^2^Department of Neuroscience, Albert Einstein College of Medicine, Bronx, NY, United States; ^3^Clem Jones Centre for Ageing Dementia Research, Queensland Brain Institute, The University of Queensland, Brisbane, QLD, Australia

**Keywords:** synapse, regeneration, synaptogenesis, synaptic homeostasis, synaptic plasticity, trafficking, membrane trafficking, vesicular trafficking

Recent advances in molecular and cellular biology have established that proteins often have more than one function, and their dominant function is highly dependent on their cellular context (Itzhak et al., 2016). Thus, protein trafficking is critical for cell biology by localizing proteins to their correct functional environment. This is especially important in neurons, where extreme cell differentiation and morphological specialization create hundreds to thousands of spatially and functionally segregated signaling domains responsible for information transfer: synapses. In neurons, trafficking and targeting of proteins and mRNA are now known to play critical roles in synaptic development, synaptic function, and plasticity, as well as maintenance. New imaging and genetic tools have revealed how both specific domains of proteins and their localization contribute or are necessary to their synaptic function (Miyazaki et al., 2021; Tullis et al., 2023). In addition to establishing baseline function, membrane trafficking plays a critical role in synaptic homeostasis and in long-range signaling from the soma to synapses and back again. Indeed, the same trafficking mechanisms are often involved in both baseline synaptic function and in various forms of synaptic plasticity, and disentangling the two is not straightforward.

The field of synaptic trafficking is broad, constantly evolving, and uses many different *in vitro* and *in vivo* models. A strong switch to *in vivo* models is now happening due to improved genetic, physiological, and imaging tools. With this topic, our goal was to gather mini-reviews and research articles from models that spanned vertebrate, invertebrate and cell culture systems, all united under the theme of how trafficking regulates synaptic function. The articles and the mini-review were relevant to broad questions in the field, such as how trafficking is regulated for synapse formation, what signaling and trafficking components are involved in development or homeostasis or both, and finally, the ways in which trafficking is dependent on synaptic or neuronal activity.

Many synaptic signaling cascades and individual proteins involved in trafficking were initially discovered as being critical for synaptic formation and neuronal development. Surprisingly, some of these same signaling cascades also play a role in degeneration. How this can be reconciled is the question that Waller and Collins aim to answer for Sarm1, Fos, and Raw using a *Drosophila* model. Their study demonstrates that the interplay of factors at multiple time points establishes either synaptic outgrowth or resilience in degeneration (Waller and Collins). A related question is posed by Parkes et al., namely, how does trafficking contribute to presynaptic protein homeostasis? They used hippocampal cell culture, *in vivo* microscopy and computational modeling to reveal that actin and microtubule vesicle transport are differentially involved in the delivery and removal of presynaptic proteins (Parkes et al.). Yet another article tackles the mechanism by which presynaptic homeostatic potentiation (PHP) at the neuromuscular junctions of *Drosophila* compensates for impaired postsynaptic glutamate receptor levels. Zhang et al. show how distinct domains of the α2δ-3 auxiliary subunit of voltage-gated calcium channels can regulate synaptic transmission and recruitment of presynaptic calcium channel abundance.

Excitatory synaptic transmission mediated by glutamate plays a central role in all nervous systems. Regulation of the trafficking of ionotropic AMPA and NMDA-type glutamate receptors has been extensively studied in the last three decades. Tightly orchestrated trafficking of glutamate receptors—from synthesis in the cell soma and travel through the ER, to motor-based long-range transport and finally to synaptic targeting and stabilization—is essential for synaptic function and neuronal plasticity. Many excellent reviews have highlighted essential proteins that regulate AMPA receptors by binding to their intracellular domains, but less has been written about those that bind to AMPA receptor extracellular domains. The mini-review by Rennich et al., addresses this, discussing how extracellular secreted proteins regulate AMPA receptor levels and their trafficking *in vitro* and *in vivo*. In addition, Chiu et al. report how the secretory trafficking protein ICA69 regulates specific plasticity-dependent AMPA receptor trafficking in the mouse hippocampus. Finally, Tan et al. describe how a resident postsynaptic protein, deubiquitinase cylindromatosis (CYLD), maintains AMPA receptors at synapses of striatal medium spiny neurons by preventing the K63-linked ubiquitination of the GluA1 and GluA2 subunits *in vivo*.

In conclusion, this topic has brought together primary research articles that shed light on new pre- and postsynaptic trafficking mechanisms essential for synapse formation, maintenance, and plasticity across *in vivo* and *in vitro* models, giving a short snapshot of how these themes are studied.

## Author contributions

FH: Writing—original draft, Writing—review & editing. PK: Writing—review & editing. VA: Writing—review & editing.
